# The Functional Role of Sphingosine Kinase 2

**DOI:** 10.3389/fmolb.2021.683767

**Published:** 2021-05-14

**Authors:** Rocio Diaz Escarcega, Louise D. McCullough, Andrey S. Tsvetkov

**Affiliations:** ^1^ Department of Neurology, The University of Texas McGovern Medical School at Houston, Houston, TX, United States; ^2^ The University of Texas Graduate School of Biomedical Sciences, Houston, TX, United States; ^3^ UTHealth Consortium on Aging, The University of Texas McGovern Medical School, Houston, TX, United States

**Keywords:** sphingolipids, sphingosine-1-phosphate, sphingosine kinase 2, nuclear lipids, aging

## Abstract

Sphingosine-1-phosphate (S1P) is a bioactive lipid molecule that is present in all eukaryotic cells and plays key roles in various extracellular, cytosolic, and nuclear signaling pathways. Two sphingosine kinase isoforms, sphingosine kinase 1 (SPHK1) and sphingosine kinase 2 (SPHK2), synthesize S1P by phosphorylating sphingosine. While SPHK1 is a cytoplasmic kinase, SPHK2 is localized to the nucleus, endoplasmic reticulum, and mitochondria. The SPHK2/S1P pathway regulates transcription, telomere maintenance, mitochondrial respiration, among many other processes. SPHK2 is under investigation as a target for treating many age-associated conditions, such as cancer, stroke, and neurodegeneration. In this review, we will focus on the role of SPHK2 in health and disease.

## Introduction

**FIGURE 1:**
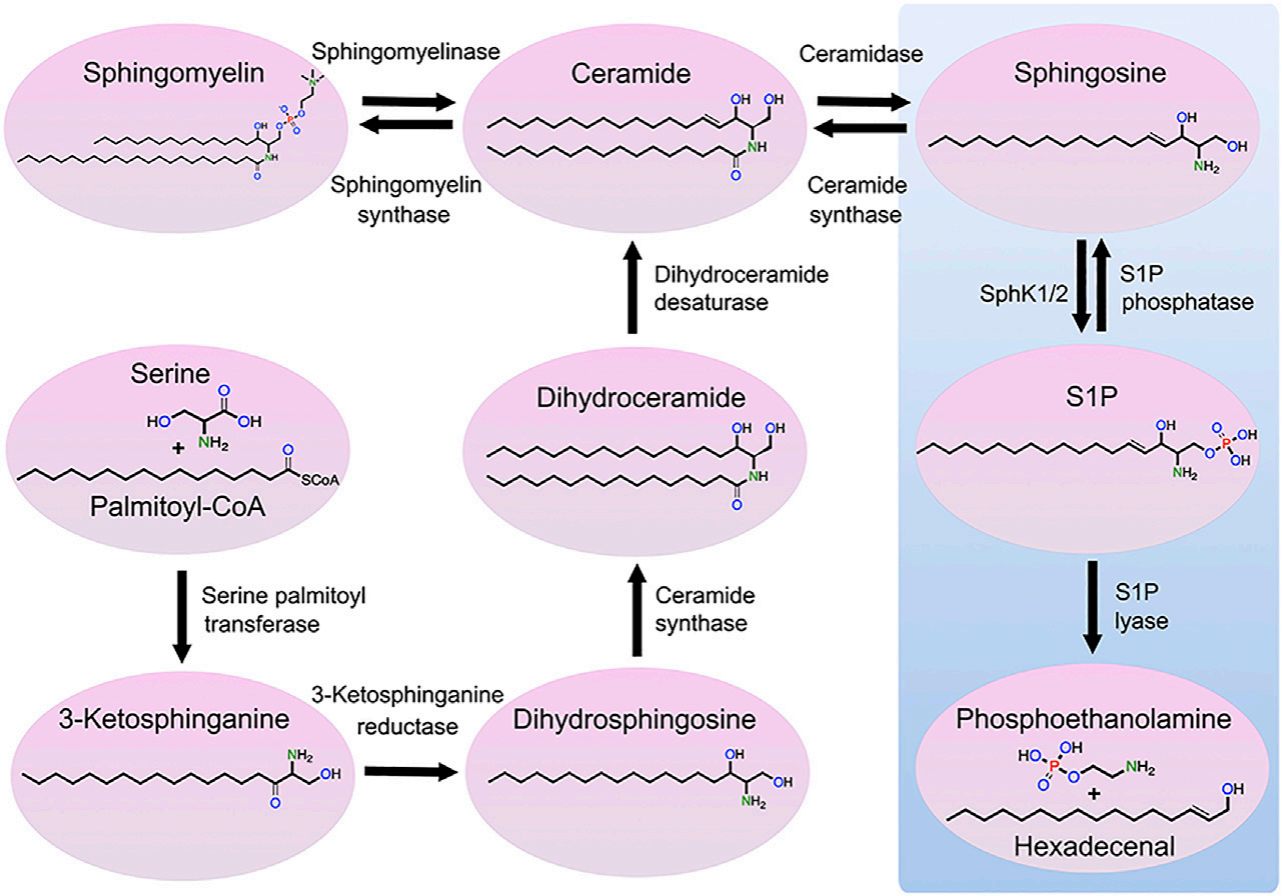
Metabolic pathway of sphingolipids. Ceramide can be generated through three mechanisms: *de novo* synthesis from serine and palmitoyl-CoA, the salvage pathway from sphingosine, and sphingomyelin hydrolysis. Ceramide can be also phosphorylated by ceramide kinase to form ceramide-1-phosphate, which can be dephosphorylated by phosphatases to form ceramide (not shown). Ceramide can be transformed into sphingosine that, in turn, can be phosphorylated by SPHK1 and SPHK2 to form S1P. S1P can be dephosphorylated into sphingosine or degraded by S1P lyase, resulting in the production of hexadecenal and phosphoethanolamine. Finally, sphingosine can be transformed to sphingadiene by the fatty acid desaturase 3 (not shown).

Sphingolipids are vital components of eukaryotic cellular membranes, which are classified according to their lipid backbone, with an octadecyl carbon chain being the most common, and a chemical modification of the lipid backbone, such as a phosphate or phosphocholine or saccharide group. Some sphingolipids, including sphingomyelins, are structural lipids of cellular membranes, whereas simple sphingolipids [e.g., sphingosine and sphingosine-1-phosphate (S1P)] are signaling lipids that modulate a wide variety of cellular processes. Numerous enzymes regulate sphingolipid synthesis and catabolism. Sphingosine kinases (SPHK) catalyze the phosphorylation of sphingosine to form S1P, changing sphingosine’s charge and considerably altering its function (Figure 1). S1P phosphatases remove the phosphate group from S1P, generating sphingosine. S1P lyase irreversibly degrades S1P to metabolites phosphoethanolamine and hexadecenal. Secreted extracellular S1P binds to the surface G-protein-coupled receptors (S1PR1-5) that modulate a large number of different cellular processes, such as platelet activation (Urtz et al., 2015).

Mammalian cells contain two sphingosine kinases. SPHK1 is present in the cytoplasm, and SPHK2 is present in several organelles, including the nucleus. Human *SPHK* genes are located on chromosomes 17 (*SPHK1*) and 19 (*SPHK2*). *SphK1* and *SphK2* exhibit some functional redundancy. *SphK1* knockout mice and *SphK2* knockout mice are viable and generally healthy, but deletion of both kinases is embryonically lethal (E13.5), indicating that S1P is a vital molecule for survival (Mizugishi et al., 2005). At least four SPHK2 isoforms have been described: the most studied is the SPHK2a isoform (Okada et al., 2005). With its extended N‐terminus, SPHK2b (Okada et al., 2005) may have greater activity than SPHK2a (Billich et al., 2003). SPHK2c and SPHK2d have been predicted but not yet discovered in cells (Alemany et al., 2007). Over the last few years, several excellent reviews about SPHK2 have been published (Neubauer and Pitson, 2013; Pyne et al., 2017; Song et al., 2018). Here, we provide a summary of our current knowledge of SPHK2 and an update of recent findings in the SPHK2 field.

## A Broad Substrate Specificity of SPHK2

The name of SPHK2 suggests that the enzyme only phosphorylates sphingosine, but in fact, SPHK2 phosphorylates several cellular and synthetic lipids. For example, d,l-threo-dihydrosphingosine and phytosphingosine are not phosphorylated by SPHK1; however, both lipids are efficiently phosphorylated by SPHK2 (Liu et al., 2000). FTY720, or fingolimod, an immunomodulatory drug used to treat multiple sclerosis, is phosphorylated by SPHK2, and SPHK2 is a primary enzyme that phosphorylates FTY720 *in vivo* (Billich et al., 2003). Lipids containing a di-unsaturated sphingadiene base accumulate in the hippocampus of *SphK2* knock-out mice. Sphingadiene-based lipids may accumulate due to lack of SPHK2-mediated phosphorylation that is supposed to be followed by catabolism of these lipids (Couttas et al., 2020). SPHK2 catalyzes phosphorylation of sphingadiene as efficiently as sphingosine (Jojima et al., 2020). Thus, SPHK2 has broader substrate specificity, and the list of lipids with the sphingoid base that can be phosphorylated by SPHK2 is expected to grow.

## Subcellular Localization of SPHK2

**FIGURE 2:**
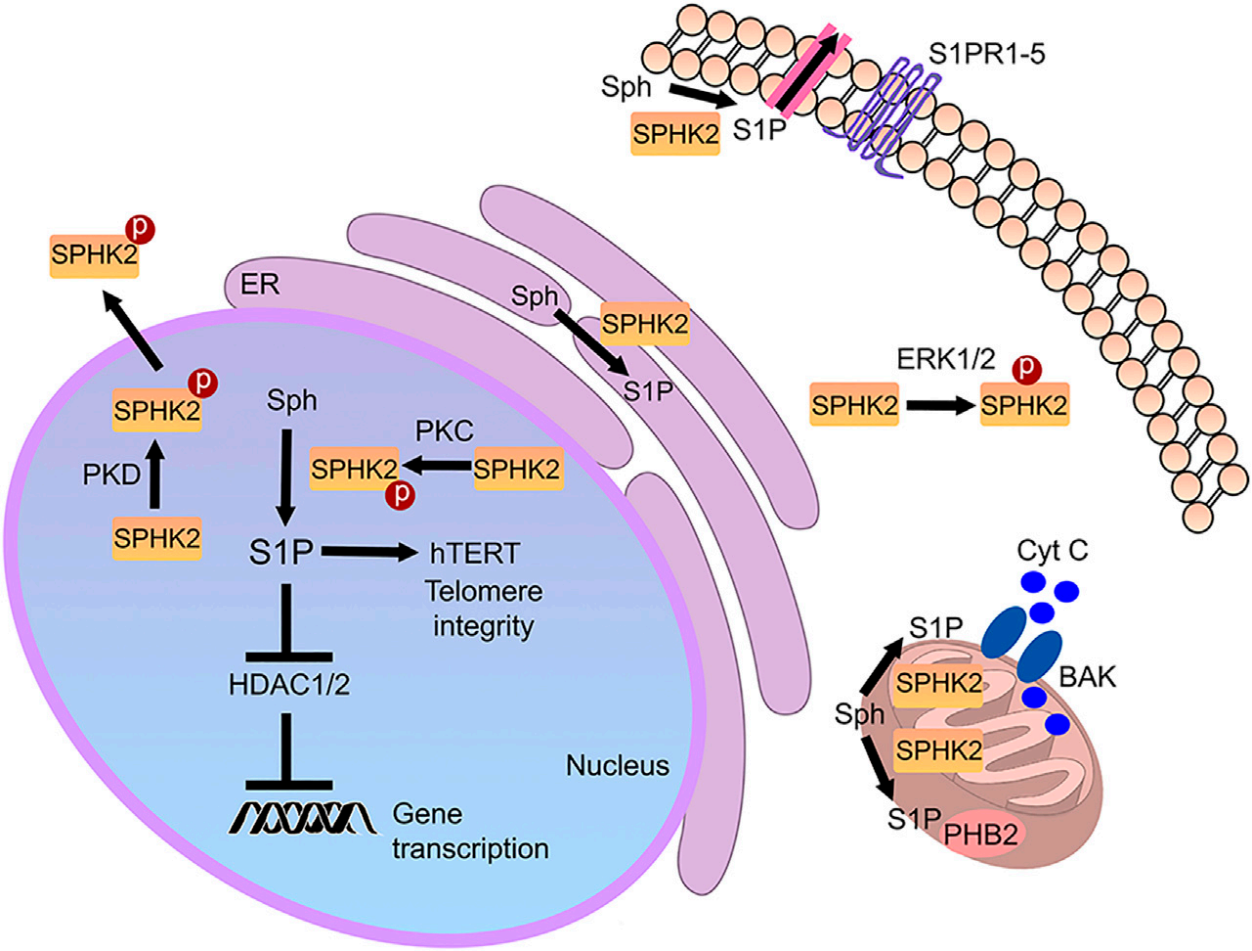
Cellular SPHK2-associated pathways. S1P generated by SPHK2 is localized to multiple sites in the cell. In the nucleus, it modulates gene expression and telomere integrity. In the mitochondria, it may regulate prohibitin 2 (PHB2) and Bax and Bak activation (BAK). In the endoplasmic reticulum (ER), it regulates cell death pathways. In cancer, SPHK2 may be recruited to the plasma membrane, where it generates S1P. Secreted or transported S1P then binds to S1PRs in an autocrine and paracrine fashion.

SPHK2 contains a nuclear localization signal and a nuclear export signal and shuttles between the nucleus and the cytoplasm (Ding et al., 2007; Figure 2). In the cytoplasm of cancerous cells and fibroblasts, SPHK2 localizes to the endoplasmic reticulum during cellular stress, where it exerts pro-apoptotic functions (Maceyka et al., 2005). In cardiomyocytes and cancer cell lines, SPHK2 is also found in mitochondria, indicating that S1P may have a role in these organelles as well (Strub et al., 2011; Sivasubramanian et al., 2015). S1P synthesized by mitochondrial SPHK2 binds prohibitin 2 (PHB2), which localizes to the inner mitochondrial membrane and regulates mitochondrial function (Strub et al., 2011). Reductions of SPHK2 levels result in defective mitochondrial respiration through dysfunctional cytochrome c oxidase (Strub et al., 2011). Finally, SPHK2 is a nuclear enzyme in many cell types, including various cancer cell types, fibroblasts, and neurons (Okada et al., 2005; Hait et al., 2009; Moruno-Manchon et al., 2017). Abnormal SPHK2 localization to the plasma membrane is linked to cancer (Neubauer et al., 2016). Thus, localizing to several organelles, SPHK2 is a multifunctional lipid kinase that modulates a variety of vital molecular mechanisms (Figure 2).

## Regulation of SPHK2

SPHK2 has basal activity towards sphingosine; however, SPHK2 can be activated by a variety of factors and conditions, including epidermal growth factor (EGF) in cancer cells Pyne and Pyne (2020) and hypoxia in cerebral microvascular endothelial cells (Wacker et al., 2009). Human SPHK2 is phosphorylated at Ser351 and Thr578 by extracellular signal-regulated kinase 1 (ERK1), and phosphorylation of these residues is important for EGF-mediated migration of cancer cells (Hait et al., 2007). In HeLa cells, phorbol 12-myristate 13-acetate promotes PKD-dependent phosphorylation of Ser419 and Ser421 in the nuclear export signal, resulting in SPHK2 export from the nucleus (Ding et al., 2007). In HEK 293 cells, PKC phosphorylates SPHK2 in the nucleus (Hait et al., 2007). In epithelial cells, PKC phosphorylates SPHK2, leading to increased histone H3 and H4 acetylation (Ebenezer et al., 2019). In a model of Huntington disease (HD), SPHK2 is hyperphosphorylated in brain samples from symptomatic mice (Moruno-Manchon et al., 2017). Phosphorylated SPHK2 levels increase in CD4^+^ T cells after lymphocytic choriomeningitis virus infection, leading to changes in gene expression (Studstill et al., 2020). In sum, SPHK2 can by phosphorylated by several kinases and appears to depend on SPHK2 localization within the cell, at least in part.

## SPHK2 and Gene Expression

In cancer cells, SPHK2 associates with HDAC1 and HDAC2 in the transcription repressor complex, and S1P generated by SPHK2 binds to and inhibits HDAC1 and HDAC2, resulting in epigenetic regulation of gene expression (Hait et al., 2009). HDAC1 and HDAC2 also regulate DNA damage responses. For example, knockdown or pharmacological inhibition of HDAC1 results in the formation of DNA double-strand breaks (DSBs) and cell death (Kim et al., 2008). HDAC1 and HDAC2 accumulate at the sites of DNA damage, which results in DNA repair, and excessive inhibition of HDACs leads to DNA damage and cytotoxicity (Kim et al., 2008). In agreement with these findings, ectopically elevating SPHK2 in the neuronal nucleus leads to enhanced histone acetylation levels, DNA DSBs, and neurodegeneration (Moruno-Manchon et al., 2017). Intriguingly, greater levels of SPHK2 are found in neuronal nuclei in Alzheimer’s disease (AD) brain samples than in control samples, suggesting that SPHK2 has a role in AD (Dominguez et al., 2018).

Although implicated in pathological conditions such as AD and HD, SPHK2 is also critical for normal neuronal function. In a model of peripheral inflammatory pain, SPHK2-deficiency reduces the expression of the P2X4 receptor that is involved in neuropathic pain, brain-derived neurotrophic factor (BDNF), and nitric oxide synthase in the spinal cord, further suggesting that SPHK2 functions as an important regulator of transcription (Canlas et al., 2015). *SphK2* ^*−/−*^ mice also exhibit lowered histone acetylation in the hippocampus, which leads to learning and memory deficits (Hait et al., 2014). In contrast, fingolimod administered to wild-type mice accumulates in the brain, including the hippocampus, where it is phosphorylated by SPHK2 and inhibits HDAC1 and HDAC2, leading to histone acetylation and expression of the genes associated with learning and memory (Hait et al., 2014).

Infection of the lung epithelium by *Pseudomonas aeruginos* *a*, a pathogenic bacterial species, leads to SPHK2 phosphorylation by PKC and its localization to the nucleus and to enhanced acetylation of histone H3 and H4, which are important for the secretion of pro-inflammatory cytokines. These results further indicate an important role for SPHK2 in gene expression (Ebenezer et al., 2019). Similarly, in cystic fibrosis airways and alveolar epithelial cells, SPHK2 is hyperphosphorylated and localized to the nucleus (Ebenezer et al., 2019). In addition, deletion of Sphk2, but not Sphk1, modulates *Pseudomonas aeruginosa*-stimulated NADPH oxidase 4 (NOX4) expression in mouse lungs, suggesting that nuclear S1P regulates NOX4 expression (Fu et al., 2021). Therefore, targeting SPHK2 is a potential strategy to mitigate inflammatory pulmonary damage in the lung.

## SPHK2 Regulates Telomeres

In the nucleus, SPHK2 has at least one other important role besides regulating gene expression. S1P synthesized by SPHK2 binds to the human telomerase reverse transcriptase (hTERT) in fibroblasts (Panneer Selvam et al., 2015). Downregulating *Sphk2* or mutating the S1P binding site in hTERT reduces the stability of the hTERT enzyme, imbalances telomere integrity, and promotes cellular senescence. Interestingly, hTERT bound to S1P can no longer bind to the makorin ring finger protein 1 (MKRN1), an E3 ubiquitin ligase that targets hTERT for degradation (Panneer Selvam et al., 2015). Inhibiting SPHK2 decreases the growth of tumor cells, and expression of wild-type hTERT, but not the S1P-binding hTERT mutant, potentiates cancerous growth, which indicates that, under some circumstances, *Sphk2* functions as a proto-oncogene (Panneer Selvam et al., 2015). The study also speculated that S1P binding to hTERT allosterically mimics hTERT phosphorylation at Ser921, preventing hTERT-MKRN1 complex formation and hTERT degradation, suggesting that SphK2 and S1P regulate a number of other nuclear proteins via protein phosphorylation mimicry.

## SPHK2 and Apoptosis

In many cell types, SPHK2 promotes cell-cycle arrest and apoptosis (Okada et al., 2005), (Maceyka et al., 2005). A few mechanisms for regulating apoptosis have been proposed. By inhibiting HDAC 1 and HDAC2, SPHK2 enhances the expression of cyclin‐dependent kinase inhibitor p21 Hait et al. (2009) and promotes DNA damage (Moruno-Manchon et al., 2017). As mentioned above, stressing cancerous cells with serum withdrawal promotes SPHK2 relocalization to the endoplasmic reticulum, where it has pro-apoptotic functions (Maceyka et al., 2005). In addition, SPHK2 localized to mitochondria may result in the activation of BAK and release of cytochrome c (Chipuk et al., 2012). Finally, SPHK2 contains a BH3 domain that appears to play a pro‐apoptotic role via interacting with the pro-apoptotic Bcl‐xL (Liu et al., 2003). Therefore, SPHK2 may promote apoptosis by multiple mechanisms.

## SPHK2 and Cell Survival

As if its functions were not complicated enough, SPHK2 seems to promote normal cell survival in many cases. Various cancer cell lines exhibit cytotoxicity or lowered proliferation and migration due to lack of the activity of SPHK2 (Magli et al., 2019). Mice lacking *SphK2*, but not *SphK1*, exhibit enhanced brain damage and poorer neurological outcomes after transient middle cerebral artery occlusion, indicating that SPHK2 is a cytoprotective lipid kinase during cerebral ischemia (Pfeilschifter et al., 2011). Similarly, *SphK2*-deficient mice suffer greater alcohol-associated liver damage (Kwong et al., 2019). *SphK2* loss suppresses hepatic insulin signaling in hepatocytes, leading to insulin resistance and glucose intolerance (Aji et al., 2020). Thus, in addition to clearly pro-apoptotic functions, SPHK2 may play a cytoprotective role.

## SPHK2 in Cell Senescence and Aging

Senescence is a homeostatic mechanism that prevents division of old or damaged cells and cancerous transformation (Trayssac et al., 2018). Besides permanent cell-cycle arrest, senescent cells undergo phenotypic changes, such as a global repression of translation, chromatin rearrangement, metabolic reprogramming, specific epigenetic modifications, morphological changes, and secretion of growth factors, cytokines, chemokines, and metalloproteinase that mediate non-cell-autonomous senescence effects (Trayssac et al., 2018). Ceramide promotes senescence, and S1P mitigates senescence phenotypes (Trayssac et al., 2018). As mentioned above, S1P synthesized by SPHK2 in the nucleus enhances hTERT stability (Panneer Selvam et al., 2015). SPHK2-deficient cancer cells and fibroblasts exhibit reduced proliferation and express senescence markers (Trayssac et al., 2018). Studies in yeast, worms, and flies showed that sphingolipids are important for regulating lifespan and aging (Trayssac et al., 2018), but the exact functions of SPHK2 in aging are not understood. Nevertheless, SPHK2 clearly plays a significant role in many age-associated diseases.

## SPHK2 and Cancer

As described above, SPHK2 is involved in tumorigenesis of diverse types of cancers. Pharmacologically inhibiting SPHK2 is beneficial in prostate, breast, ovarian, pancreatic and kidney, colon, liver, blood, and lung cancers (Pyne and Pyne, 2020). Various SPHK2 inhibitors are being developed to treat cancer, including SPHK2-specific sphingosine‐competitive small molecules (Magli et al., 2019). Two excellent reviews about the S1P signaling in cancer were recently published (Pyne and Pyne, 2020; Pitman et al., 2021).

## SPHK2 and Inflammation

While SPHK1 is a clearly pro-inflammatory enzyme, the roles of SPHK2 in inflammation are not completely understood. Data generated with SphK2^−/−^ mice show that SPHK2 might be an anti-inflammatory lipid kinase (Pyne et al., 2017). However, SPHK2 inhibitors suggest that it is a pro-inflammatory kinase. The picture is also complicated by an increase in SPHK1 expression in *SphK2* ^*−/−*^ mice (Pyne et al., 2017). For example, SPHK2 is important for resolving inflammatory vascular lung injury (Joshi et al., 2020). However, pharmacologically inhibiting SPHK2 leads to activation of anti‐inflammatory mechanisms in models of Crohn’s disease Maines et al. (2010) and renal inflammation (Schwalm et al., 2021). Conversely, in a model of collagen-induced arthritis, siRNA-mediated SPHK2 downregulation leads to higher levels of proinflammatory cytokines and more aggressive disease than in control mice (Lai et al., 2009). Genetic SphK2 deficiency exacerbates the formation of atherosclerotic lesions in a mouse model of atherosclerosis (Ishimaru et al., 2019). Therefore, pharmacologically inhibiting and genetically downregulating SphK2 often lead to different, occasionally opposing effects, which could be explained by SPHK1-associated compensatory mechanisms, including increased circulating S1P. SPHK2 inhibitors may also influence on several cellular pathways that synergistically result in anti‐inflammatory outcomes.

Two recent studies, however, suggest that inhibiting or deleting SphK2 leads to the same outcomes. For example, SphK2 is a negative regulator of macrophage activation; inhibiting SPHK2 or deleting SphK2 in mouse peritoneal macrophages increases lipopolysaccharide-induced inflammatory cytokine production (Weigert et al., 2019). Likewise, inhibiting SPHK2 or deleting SphK2 in a mouse model of renal inflammation and fibrosis decreases inflammation and fibrotic responses, resulting in lessened renal injury (Ghosh et al., 2018).

## SPHK2 and Neurodegenerative Diseases

The roles of SPHK2 in neurodegeneration have only begun to be investigated. SPHK2 activity declines in the hippocampus and temporal cortex of AD patients, although the significance of this finding is not clear (Couttas et al., 2018). Another study reported elevated levels of nuclear SPHK2 in AD, suggesting that SPHK2 is involved in transcription in AD (Dominguez et al., 2018). Deletion of *SphK2* in a mouse model of AD reduces Aβ deposition and hippocampal epileptiform activity (Lei et al., 2019). However, *SphK2* deletion also results in hippocampal volume loss, myelin and oligodendrocyte reduction, and exacerbates memory deficits (Lei et al., 2019).

In multiple sclerosis, FTY720, a pro-drug, acts as an immunosuppressive factor subsequent to its phosphorylation by SPHK2 and modulation of S1PR1 (Billich et al., 2003). In a neurotoxin-induced model of Parkinson’s disease (PD), SPHK2 is downregulated in the substantia nigra, where it is present in mitochondria of dopaminergic neurons (Sivasubramanian et al., 2015). Levels of SPHK2 are not altered in HD human brains Di Pardo et al. (2017) and in brains of symptomatic HD mouse models, YAC128 and BACHD mice (Di Pardo et al., 2017; Moruno-Manchon et al., 2017). Cortical brain samples from the R6/2 mouse model, an aggressive mouse model of HD, exhibit increased SPHK2 levels in fully symptomatic mice but no increase in early manifest mice (Di Pardo et al., 2017). Pharmacologically targeting SPHK2 has been proposed as a therapy in HD (Moruno-Manchon et al., 2017; Di Pardo and Maglione, 2018). Overall, the SPHK2/S1P pathway is under investigation in several age-associated neurodegenerative diseases, but it is still necessary to learn if changes in the pathway in these disorders are a cause or an effect of neuronal malfunction.

## Sex Differences and SPHK2

Lipid signaling pathways are often sex-specific. Sex-associated differences in sphingolipid metabolism are found in the aging human brain. With liquid chromatography tandem mass spectrometry, ceramide, sphingomyelin, and sulfatide levels are correlated with age in the hippocampus of males (Couttas et al., 2018). Intriguingly, S1P normalized to sphingosine is inversely correlated with age in females, suggesting that reduced S1P has a negative role in cell senescence and female vulnerability in AD (Couttas et al., 2018). Sphingadienine levels are sex-associated being on average 30% higher in females, which appears to depend on the fatty acid desaturase 3 (Karsai et al., 2020). Direct involvement of SPHK2 in sex-differences remains to be shown; nevertheless, pharmacologically inhibiting SPHK2 leads to downregulation of estrogen-dependent signaling pathways (Antoon et al., 2010). Thus, as at least some of lipid signaling pathways are sex-associated, lipids in general and SphK2/S1P in particular may play important roles in many sex-associated diseases.

## Conclusions and Future Directions

SPHK2 is a multifunctional lipid kinase, which functions are far from being understood. SPHK2 phosphorylates different intracellular sphingosine pools, leading to diverse effects and often opposing cellular fates. Although SPHK2 may be cytoprotective in some cancers, research has focused on developing small-molecule SPHK2 inhibitors that kill malignant cells or inhibit cancer growth. Pharmacological inhibition SPHK2 restricts inflammation, which has widespread ramifications for a number of inflammatory diseases. As S1P modulates cell senescence, understanding roles of SPHK2 in senescence of different cell types is an exciting research field. Much is still unknown about the role of SPHK2 in the brain. SPHK2 is clearly involved in neurodegenerative diseases, but whether and how SPHK2 regulates the initiation and progression of AD, PD, HD, and other neurodegenerative diseases has not yet been thoroughly investigated. These will be important questions for future research.
